# Metabolic Interactions in the Gastrointestinal Tract (GIT): Host, Commensal, Probiotics, and Bacteriophage Influences

**DOI:** 10.3390/microorganisms3040913

**Published:** 2015-12-17

**Authors:** Luis Vitetta, Sean Hall, Samantha Coulson

**Affiliations:** 1 Medlab Clinical Ltd., Sydney 2015 Australia; E-Mails: sean_hall@medlab.co (S.H.); samantha_coulson@medlab.co (S.C.); 2 Sydney Medical School, University of Sydney, Sydney 2006, Australia

**Keywords:** bacteria, metabolite signaling, dysbiosis, gastrointestinal tract

## Abstract

Life on this planet has been intricately associated with bacterial activity at all levels of evolution and bacteria represent the earliest form of autonomous existence. Plants such as those from the *Leguminosae* family that form root nodules while harboring nitrogen-fixing soil bacteria are a primordial example of symbiotic existence. Similarly, cooperative activities between bacteria and animals can also be observed in multiple domains, including the most inhospitable geographical regions of the planet such as Antarctica and the Lower Geyser Basin of Yellowstone National Park. In humans bacteria are often classified as either beneficial or pathogenic and in this regard we posit that this artificial nomenclature is overly simplistic and as such almost misinterprets the complex activities and inter-relationships that bacteria have with the environment as well as the human host and the plethora of biochemical activities that continue to be identified. We further suggest that in humans there are neither pathogenic nor beneficial bacteria, just bacteria embraced by those that tolerate the host and those that do not. The densest and most complex association exists in the human gastrointestinal tract, followed by the oral cavity, respiratory tract, and skin, where bacteria—pre- and post-birth—instruct the human cell in the fundamental language of molecular biology that normally leads to immunological tolerance over a lifetime. The overall effect of this complex output is the elaboration of a beneficial milieu, an environment that is of equal or greater importance than the bacterium in maintaining homeostasis.

## 1. Introduction

**Figure 1:**
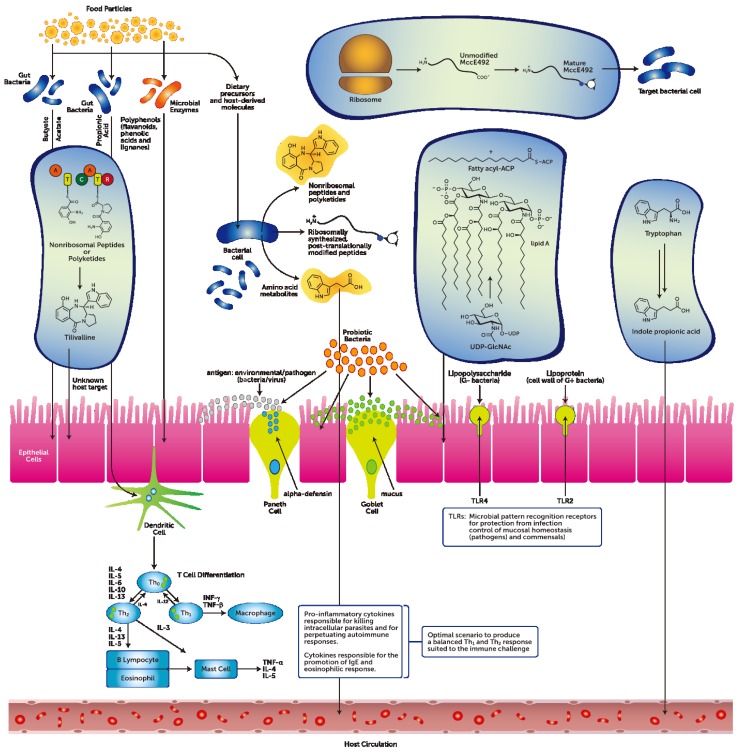
A diagrammatic representation of the complex interactions that are elaborated in the gastrointestinal tract by host-microbe and microbe-microbe interactions in order to maintain homeostasis. Sourced from (Donia and Fischbach, 2015 [10]; Takeda and Akira, 2015 [11]; Jiménez-Dalmaroni *et al.*, 2015 [12]; Arpaia and Rudensky, 2014 [13]; Russell *et al.*, 2013 [14]; Shimada *et al.*, 2013 [15]).

Bacteria that live on and within humans outnumber human cells by a factor of 10 [1]. It is estimated that 10^14^ bacteria populate the human gut and this active cohort is separated from the mucosal layer of the intestines by a single layer of intestinal epithelial cells; the bacteria, however, do not come into direct contact with the epithelial cells under normal physiological conditions, but rather remain in the lumen or outer mucus layer [2]. Moreover, there is also a secretion of mucin (mucus) layer (single layer in the small bowel and multi-layered in the large bowel) elaborated by the goblet cells. The mucin is largely composed of glycosylated proteins that serve an important luminal protective function throughout the GIT, covering the apical surfaces of the enterocytes [3] (Figure 1).

The bacteria in the GIT are subject to two distinct but related trends that exemplify the bacterial complexity that exists therein. Firstly, there is an increasing bacterial density that occurs from the proximal GIT (e.g., stomach) to the distal GIT (e.g., the large bowel). The second trend is the increasing complexity and diversity of the bacterial species that occur in the same GIT proximal to distal direction. Most of the bacterial species are GIT lumen residents, whereas fewer but well established proteobacteria including *Akkermansia muciniphila* reside and perpetuate within the mucus layers close to the epithelial tissue [4]. Other bacteria, such as the segmented filamentous bacteria (SFB) [5], are indigenous GIT commensal bacteria that interact directly with the intestinal epithelial cells in the terminal ileum [6]. In contrast to those gut bacteria that are classified as invasive pathogens (e.g., *Shigella* sp.), SFB do not invade epithelial cells, penetrate the epithelial barrier, or induce intestinal inflammation [5] that may disrupt the epithelial barrier and its functionality. By contrast, SFB that are present in the microbiota from Th17 cell-deficient and Th17 cell-sufficient mice have been demonstrated to be capable of specifically inducing Th17 cells in the GIT. As such, SFB represent the first example of a commensal species that can skew the mucosal effector T cell balance and thus affect the immune fitness of the individual [7].

The advent of the human microbiome project has encouraged a new perspective on bacteria that live on and within humans [8]. A complex profile between human host and bacteria emerges, as demonstrated by the greater gene contribution by the microbial cohort that is of the order of 8–9 million protein coding genes (of which approximately one third reside in the GIT) *versus* the current 20–25,000 human protein coding genes that have been identified [9]. As such, bacteria and the host elaborate a multitude of chemical compounds that have a fundamental role in the maintenance of homeostasis of host-microbe and microbe-microbe physiology (Figure 1).

## 2. What this Review Proposes to Add

The view that the gastrointestinal tract (GIT) is a collection of toxic waste and is largely an inert organ has been significantly challenged over the last decade. This review presents an overview of the research that has demonstrated that the GIT microbiota provide essential metabolic and physiological functions for human survival such as the harvesting of essential food nutrients, vitamins and energy; metabolism of xenobiotics; protection from opportunistic pathobionts (pathogenic capabilities); influencing neurological pathways; development and maturation of the intestinal epithelium; and the development and maintenance of homeostasis of localized immune function. In this respect and in a neonate, the importance of microbial metabolic activity is evidenced via the assault of all mucosal surfaces and the skin, where the bacteria that colonize the GIT provide essential cues for the development of immunological tolerance and metabolic homeostasis over a lifetime for the host.

The complexity of host-microbial and microbial-microbial metabolite activities that influence the homeostasis of host immune and metabolic tissues in health and disease is beyond the scope of any one review. Therefore, for an in-depth appraisal we refer the reader to additional relevant reviews at the end of each of the following sections. 

## 3. The Gastrointestinal Tract Barrier and Chemical Transmitters

There are recognized variations in the cell-to-cell junctions that bind GIT epithelial cells together. In the stomach and the large bowel the junctions form a tight barrier so that very small quantities of solutes can pass between the cells [16,17]. In the small intestine though, the cell-to-cell junctions are not as tight [17]. The intestinal epithelium in this anatomical area is considered *leaky* because some water and solutes can be absorbed between the cells (via the selective paracellular permeability pathway) instead of through the enterocyte. Furthermore, on the serosal (basolateral area) surface of the epithelium, substances being absorbed from the lumen and molecules secreted by epithelial cells enter the extracellular fluid. The mucosa of the GIT expresses the most adaptable characteristic of the GIT—it varies extensively from region to region of the GIT (*i.e.*, from the stomach to the small bowel to the large bowel). The cells of the mucosa include the transporting epithelial cells-enterocytes in the small bowel, endocrine, and exocrine secretory cells, as well as stem cells. At the mucosal or apical surface of the epithelium cell there ions, enzymes, mucus, and paracrine molecules are secreted into the lumen; the latter describes paracrine signaling. Diffusion of these molecules occurs over short distances and influences the function of adjacent cells in the GIT when the appropriate receptors are present, inducing a response [18,19]. There is a complex network of cellular communication that has been established in the GIT and it is comprised of chemical transmitters that in the gut have been reported to be produced by discrete cells of the GIT mucosa; these have been classified as endocrine, paracrine, synaptic, or autocrine molecules [20,21]. Further, more than 30 gut hormone genes and a plethora of bioactive peptides have been reported, making the GIT the largest and most complex endocrine site [21]. Also there is a highly conserved set of receptors and pathways that include the fibroblast growth factor family, Hedgehog family, Wnt family, and TGF-β superfamily. Binding of a paracrine factor to the specific receptor initiates signal transduction cascades that elicit different metabolic responses. The initial stimulus for hormone or other peptide molecule secretions is the ingestion of food. Moreover, it should be noted that there is a diverse set of hormones and transmitters that is elaborated by the GIT mucosal epithelium [22]; these chemical messengers from the GIT can have far-reaching effects throughout the body, for example influencing end organs such as the brain and the liver [23]. 

It is known that the paracellular semi-permeable junctions have a characteristic plasticity and that the *tightness* and *selectivity* can be regulated to some extent [24]. The gastrointestinal tract consisting of a single epithelial layer is a functional unit that provides a physical barrier and accomplishes a legion of tasks from protection against pathogen invasion to nutrient assimilation and absorption, mucus secretion, and the maintenance of immune function homeostasis by control of pro- and anti-inflammatory activity [25]. It has also been posited that GIT barrier disruption by environmental/nutritional/pathogenic triggers can disturb end-organ physiology [23,26]. Therefore minimizing its disruption whilst maintaining the GIT barrier function is of primary importance in gut health and end-organ physiological homeostasis. Moreover, mechanistically tight junctions in the GIT are of critical importance for the establishment and maintenance of gut cell polarity [24]. This is important as it drives injury repair of the GIT barrier [27], given the extensive and complex interactions with microbes, environmental, and dietary-related antigens.

A recent review has posited that the gut-liver-lung axis may play a significant modifying role in the pathogenesis of chronic obstructive pulmonary disease [28]. The beneficial effect of dietary fiber on lung function was cited as being due to modulation of innate immunity and subsequent attenuation of the pulmonary response to inflammatory stimuli. This effect may be gut triggered. A murine study partly confirms this notion by reporting that the intestinal microbiota exerted a protective role during pneumococcal pneumonia [29]. In that study it was reported that the GIT microbiota was deemed to enhance alveolar macrophage function.

Another murine study that investigated the metabolism of dietary fiber by the GIT microbiota demonstrated that dietary fermentable fiber content changed the composition of the gut and lung microbiota, specifically by altering the ratio of *Firmicutes* to *Bacteroidetes* [30]. This study reported that (i) the gut microbiota metabolized the fiber, consequently increasing the concentration of circulating short-chain fatty acids (SCFAs); (ii) the mice that had been fed a high-fiber diet had increased circulating levels of SCFAs and were protected against allergic inflammation in the lung; (iii) an alternative low-fiber diet showed decreased levels of SCFAs and increased allergic airway disease. Mechanistically, the study reported that when the mice were treated with the SCFA propionate it altered bone marrow hematopoiesis characterized by enhanced generation of macrophage and dendritic precursor cells. Moreover, that subsequent seeding of the lungs by dendritic cells with high phagocytic capacity had an impaired ability to promote Th2 cell effector function. The molecular mechanism that linked propionate to allergic inflammation was dependent on the G protein-coupled receptor 41 (GPR41) but not GPR43; further, the results demonstrate that dietary fermentable fiber and SCFAs can shape the immunological environment in the lung and influence the severity of allergic inflammation.

For an in-depth appraisal of this section see [10,31,32,33].

### 3.1. Diet-Independent Metabolites from the GIT Microbiota

The metabolites encountered in the GIT can be products of microbial metabolism. Further, metabolites can be produced during growth and these may then lead to the production of secondary as well as co-metabolites [10,14,34].

Experiments with germ-free mice models have clearly demonstrated that colonization with commensal bacteria provides the essential cues for the initial development and maturation of the mammalian immune system [35]. In studies with germ-free mice that have been reared in sanitized environments, the animals express immature gut-associated lymphoid tissues [36], reduced production of IgA [37], and underproduction of T regulatory lymphocytes in the large bowel [38]. Microbial derived molecules have been reported to at least partly mediate the immunomodulatory response [35]. 

Diet-independent bacterial products predominantly consist of bacterial cell wall components such as peptidogycans and lipopolysaccharides that can prompt a response through the activation of Toll-like receptor 4 (TLR4)-dependent and nucleotide binding oligomerization domain receptor-dependent signaling pathways (Figure 1) [39]. Also, the commensal/opportunistic pathobiont *Bacteroides fragilis* produces a diet-independent immunomodulatory molecule, namely lipopolysaccharide A, that activates TLR4. Lipopolysaccharide is a major component of the outer membrane of Gram-negative bacteria and shows potent immune-stimulatory activity [11].

The administration of polysaccharide A to germ-free mice has been reported to trigger T-cell growth in the spleen, rescuing a T_H_1-T_H_2 imbalance that is skewed toward T_H_2 [40]. In the GIT immune system polysaccharide A has been shown to increase IL-10 production by CD4^+^ T cells and to reciprocally suppress IL-17A responses via TLR2 [41]. Furthermore, evidence from numerous studies has reported that TLR2 can recognize lipoprotein components from a variety of microbial pathogens such as *Mycoplasma fermentans*, *Treponema pallidum*, *Borrelia burgdorferi*, peptidoglycan and lipoteichoic acid from gram-negative bacteria; lipoarabinomannan from mycobacteria, glycosyl-phophatidyl-inositol anchors from *Trypanosoma cruzi*, a phenol-soluble modulin from *Staphylococcus epidermis*, zymosan from fungi, and glycolipids from *Treponema maltophilum* [11]. Diet-independent, microbial-generated metabolites have distinct roles in GIT homeostasis preservation and that there is also co-operation between TLR receptors that as such can coordinate and fine-tune an immunomodulatory response [11].

For an in-depth appraisal of this section see [11,12,13,14,15,42].

### 3.2. Diet-Dependent Metabolites from the GIT Microbiota

Quorum-sensing has been defined as a cell-to-cell signaling mechanism that enhances the ability of bacteria to respond to chemical molecules; these are designated as auto-inducers because they can elicit a molecular response. Indole is such a molecule and is produced from the metabolism of tryptophan by the enzyme tryptophanase, which is elaborated by a variety of Gram-positive and Gram-negative GIT bacteria [43]. In the mammalian GIT, indole has been reported to regulate numerous microbial activities that include biofilm formation in the gut, antibiotic resistance, bacterial motility, and host cell invasion [43]. Furthermore, indole has also been reported to augment epithelial barrier functions both from *in vitro* and *in vivo* studies by upregulating the components of the tight junction complexes [15,44]. This is important because intercellular tight junctions tightly regulate paracellular antigen trafficking [45]. Also, given that tight junctions are extremely active structures depending on their anatomical locales (e.g., the small bowel, the large bowel), they can operate in several key functions of the intestinal epithelium under both physiological and pathological circumstances. As such, intestinal permeability, together with antigen sampling by enterocytes and luminal dendritic cells, the regulation of molecular trafficking between the intestinal lumen, and the sub-mucosa/mucosa, is paramount in order to maintain homeostasis, as delineated by tolerance and immunity to non-self [46].

There are also present, especially in the GIT, diet-dependent metabolites that are either elaborated by the host or the microbial cohort (Figure 1). Diet-dependent metabolites have been reported to influence the GIT epithelial barrier and immune regulation as well as pro- and anti-inflammatory activity. These metabolites include short and long chain fatty acids (e.g., acetate, propionate, butyrate), vitamins, and bile salts [35,47]. The anti-inflammatory effects of short chain fatty acids (SCFAs) has been well documented in recent times [48,49]. An early report on the wide-ranging effects of SCFAs posited that there was a concentration gradient dependence that was relative to tissue-specific physiological functions. This SCFAs gradient was demonstrated to vary from the total SCFAs detected in the GIT lumen (*i.e.*, ~100 mM) to that in the portal blood (*i.e*., ~400 μM) and to that in the peripheral blood (*i.e.*, ~100 μM) [50]. A recent review [31] has documented the diverse effects that dietary-GIT, microbial-derived SCFAs can have on the regulation of host immune and metabolic tissues.

In GIT-associated immune tissues, SCFAs have been shown to have a positive impact on the terminal differentiation of CD4^+^ helper T cells [48]. Studies have shown that the administration of single or multiple SCFAs formulations to germ-free or specific antigen-free mice increased T regulatory cells [42,51], which is a central component of mucosal tolerance. Also, it has been shown that SCFAs have a central role in the suppression of inflammation and allergic immune responses [52]. Further investigations have shown that the effect of SCFAs on T regulatory cell induction is not shared by all SCFAs. Among SCFAs, butyrate demonstrates the strongest effect for native T cell differentiation into T regulatory cells [53]. Moreover, butyrate indirectly induces IL-10 into producing T regulatory cells that communicate anti-inflammatory activity to dendritic cells. Propionate also shows a T regulatory cell differentiation inducing effect, but to a lesser extent, whereas acetate has no impact on T regulatory cell differentiation. Hence it has been hypothesized that locally produced butyrate in the GIT is the principal trigger for T regulatory cell differentiation and that orally administered acetate and propionate may be important factors for the migration of T regulatory cells into the colon [53]. Niacin has also been reported to have a similar anti-inflammatory effect [54]. Therefore these outcomes clearly show that microbial-derived molecules such as SCFAs and niacin provide important molecular cues in the maintenance of gut immune tissue homeostasis by triggering increased T regulatory cell proliferation and accumulation and the control of a balanced pro-/anti-inflammatory response. 

The molecular mechanism that links the effects of butyrate on immune cells has been reported to be mediated by G protein-coupled receptors [31], these being the most versatile receptor family reported in that they have the ability to respond to chemically diverse ligands [55]. The major SCFAs receptors are Gpr41 and Gpr43, which are both expressed on immune cells [56]. In a murine study of induced colitis it was reported that SCFAs (mainly acetate and propionate and to a lesser extent butyrate) bind Gpr43 and that SCFAs-G protein-coupled receptor interactions were reported to profoundly affect inflammatory responses [48]. Moreover, it was also reported that stimulation of Gpr43 by SCFAs was a necessary molecular signal that resolved certain inflammatory responses. This was further supported by observations that showed that Gpr43-deficient mice displayed exacerbated or unresolved inflammation in models of colitis, arthritis, and asthma [48,57]. This result was postulated to be related to the increased production of inflammatory mediators by Gpr43-deficient immune cells and increased immune cell recruitment. Furthermore, germ-free mice that lack bacteria express few or no SCFAs showing dysregulation of inflammatory responses. The combined results of these experiments indicate that Gpr43 binding of SCFAs provides a molecular link between dietary and gastrointestinal bacterial metabolic cues linked to immune and inflammatory responses in order to maintain homeostasis.

In skeletal muscle and the liver, butyrate has been shown to enhance fatty acid oxidation and thermogenesis by increasing the expression of peroxisome proliferator-activated receptor-gamma coactivator-1α (PGC-1α) and the phosphorylation of adenosine-monophosphate-activated kinase (AMPK). Butyrate has also been shown to enhance the expression of PGC-1α and mitochondrial uncoupling protein-1 in brown adipose tissues [58]. Furthermore, butyrate with propionate can activate intestinal gluconeogenesis via a gut-brain neural circuit that has been reported to promote metabolic benefits on body weight and glucose control [59].

SCFA metabolites such as acetate and propionate produced by GIT microbes that bind Gpr43 and Gpr41 receptors enhance gut epithelial barrier functions [60]. The Gpr43 and Gpr41 receptors are expressed on intestinal endocrine L-cells, where the release of PYY and GLP-1 peptides links this metabolic activity to energy homeostasis. In addition, Gpr41 is also expressed in sympathetic ganglia [61]. Propionate activation of Gpr41 increases heart rate and energy expenditure through sympathetic activation as well as norepinephrine release from sympathetic neurons [62]. Hence sympathetic activity is regulated through sensing nutritional states, the net effect being the maintenance of energy homeostasis.

A butyrate and beta-hydroxybutyrate specific receptor Gpr109A [31,55,63] has been reported to suppress colonic inflammation and carcinogenesis by promoting anti-inflammatory properties in colonic macrophages and dendritic cells [54]. The anti-inflammatory activity induces the differentiation of regulatory and IL10-producing T cells. GPR109A is also expressed in adipose tissues and activated adipose tissue macrophages, where it regulates lipid homeostasis [63].

The commensal *Clostridia* group [64] consists of Gram-positive, rod-shaped bacteria within the phylum *Firmicutes*; these make up a substantial part of the total bacteria in the GIT microbial cohort. Atarashi and colleagues [65] have shown in a murine model that the spore-forming component of the indigenous intestinal microbiota, particularly clusters IV and XIVa of the genus *Clostridium*, promoted T regulatory (Treg) cell accumulation and that the Treg cells were most abundant in the colonic mucosa. It was also demonstrated that oral inoculation with *Clostridia* during the early life of conventionally reared mice resulted in resistance to colitis and systemic immunoglobulin E responses in adult mice, strongly indicating that these commensals play a critical role in the maintenance of immune homeostasis. Hence the CD4+ Treg expressing the Foxp3 transcription factor were posited to play a critical role in the maintenance of immune homeostasis. An additional study from this group [38] investigated manipulating the GIT microbiota in an animal model. After the isolation of CD4+ FOXP3+ Treg-cell-inducing bacterial strains from a healthy human indigenous microbiota, mice were colonized with this fecal sample. Subsequently, 17 strains from these mice were isolated on the basis of possessing high potency in enhancing Treg cell abundance and inducing important anti-inflammatory molecules—such as IL-10 and inducible T-cell co-stimulator (ICOS)—in Treg cells upon inoculation into germ-free mice. Genome sequencing revealed that the strains fell within clusters IV, XIVa, and XVIII of *Clostridia* and lacked prominent toxins and virulence factors. It was concluded that the combined 17 strains acted as a community to provide bacterial antigens and a TGF-beta-rich environment to assist with the expansion and differentiation of Treg cells. Further, Treg induction from the fixed mixture of 17 *Clostridia* strains from the human microbiota in the mice attenuated disease models of colitis and allergic diarrhea.

In studies with germ-free mice incubated with pathogenic *Escherichia coli* O157:H7 the animals succumb to a lethal infection [66]. In additional studies when germ-free mice were inoculated with *Bifidobacteria* strains, the enterohemorrhagic *E. coli* O157-induced death was prevented [57]. This study [57] demonstrated that acetate derived from specific *Bifidobacteria* strains inhibited the translocation of luminal Shiga toxin from the gut lumen to the blood by improving epithelial barrier defenses and functionality as well as suppressing colonic inflammation. These findings provide insight into the mode of action through which probiotic bacteria exert a protective effect against pathological infections, and further proposes new insights into how bacterial metabolites may regulate the epithelial barrier and its functions. 

Hence metabolites from the intestinal microbiota cohort provide key molecular determinants of host-microbiota mutualism that consequently may influence health or disease in the intestinal tract. What is less understood is whether the host-microbiota molecular crosstalk can influence inflammation in peripheral end organ tissues such as the liver or the lungs. To this effect, recently it was reported that GIT-derived SCFAs (e.g., propionate) alleviated an allergic airway response that was triggered by a house dust-mite extract in a murine model [30]. Furthermore, omega-3 polyunsaturated fatty acids and ursodeoxycholic acid have been reported to have an additive effect in attenuating a diet-induced non-alcoholic steatohepatitis in mice [67]. This perhaps is associated with GIT microbial cohort crosstalk, which was triggered by the administered compounds.

For an in-depth appraisal of this section see [15,55,64,68,69].

### 3.3. The Metabolic Effects of Probiotics

A current unified definition of probiotics states that these are live microorganisms that, when administered in adequate amounts, confer a health benefit on the host [70]. The variety of probiotics, including those from such genera as the *Lactobacilli*, *Bifidobacteria*, *Escherichia coli*, *and Streptococci* or from the yeast *Saccharomyces*, presents a complex picture that is as multifaceted as their clinical applications [23]. 

Studies have reported that probiotic bacteria that can stabilize the GIT barrier function do so by instructing the mucosal barrier to generate anti-microbial peptides such as defensins [71] (Figure 1). Moreover, probiotic bacteria can exert control over immune responses and cellular proliferation [23]. Specifically, probiotic bacteria from the genera *Lactobacilli* have been reported to induce human enterocyte beta-defensin 2 [72]. The mechanism that has been postulated is the induction of pro-inflammatory pathways that includes NFkB, AP-1, and MAPKs [72]. Similarly, the induction of human enterocyte beta-defensin 2 has also been reported for the *Escherichia coli* Nissle 1917 strain, mediated though the probiotic’s flagellin [72]. Additional experiments with *Lactobacillus brevis*-derived polyphosphate reported that it enhanced the GIT epithelial barrier function and maintained intestinal homeostasis [73]. This was achieved through the integrin-p38 MAPK pathway.

The mucus layer covering the GIT serves as an important restrictive barrier to microbes and is the contact point with most of the GIT microbiota in the human host [23]. A recent study elucidating the structural and molecular insights into probiotic bacteria adherence to the mucus layer reported on a unique cell-surface protein, Lar0958, from *Lactobacillus* *reuteri* that mediated adhesion of *L. reuteri* human strains to the GIT mucus [74].

The formation of low molecular weight compounds (*i.e.*, less than 1,000 Da), such as organic acids (e.g., lactic acid), and the production of antibacterial substances, namely bacteriocins (*i.e.*, greater than 1000 Da) have been postulated as compounds that can afford health benefits [75]. Organic acids such as acetic acid and lactic acid have a reported strong inhibitory effect against Gram-negative bacteria, and have been considered the leading antimicrobial compounds responsible for the inhibitory activity of probiotics against pathogenic bacteria [75]. Furthermore, many *Lactobacilli* species produce a range of antibacterial peptides that include bacteriocins and small antibacterial peptides [76]. The bacteriocins produced by lactic acid bacteria include lactacin B (*L. acidophilus*), plantaracin (*L. plantarum*), and nisin (*Lactocccus lactis*); these have been reported to have a low range of activity while acting against closely related bacteria [77,78].

Antibacterial compounds have also been reported for a number of *Bifidobacteria.* A specific bacteriocin termed bifidocin B, produced by *B. bifidum* NCFB 1454, has been described as active against Gram-positive bacteria [75]. Additional studies have detected an unidentified low molecular weight lipophilic molecule (from the *Bifidobacteria* strains) that showed strong killing activity against pathogenic bacteria such as *Salmonella enterica ser. typhimurium* SL1344, and *E. coli* C1845 [79]. Also, a low molecular weight protein (*i.e.*, designated as BIF and elaborated by *B. longum* BL1928) has been characterized as having anti Gram-negative activity [80]. Probiotic bacteria have also been reported to have the capacity to produce bile acid derivatives such as de-conjugated bile acids. De-conjugated bile acids demonstrate antimicrobial activities [81]. Furthermore, probiotic bacteria have also been reported to produce metabolites that inhibit the growth of fungal species and these compounds include benzoic acid, methylhydantoin, and mevalonolactone [82,83]. Recently we have presented data from a murine model of a high fat diet (HFD)-induced non-alcoholic fatty liver disease (NAFLD) that demonstrated that HFD induced local GIT dysbiosis by disrupting the integrity of the intestinal epithelial barrier [84]. The results showed that mice fed a HFD followed by the administration of a multi-species probiotic formulation maintained tight junction proteins Zonulin-1 and Zonulin-2 (*i.e.*, decreased intestinal epithelial cell permeability) and reduced hepatic triglyceride concentrations compared to mice fed the HFD alone. Furthermore, the study demonstrated that supplementation with a multi-species probiotic preparation non-significantly reduced the effects of a HFD by attenuating the progression of steatosis/NAFLD, as evidenced in part from liver tissue histology, with 60% of the mice showing visible reductions in fat droplets. The mechanistic posit that was advanced in that study was consistent with those studies that have reported a beneficial effect with the introduction of probiotics to improve high fat diet-promoted NAFLD [85].

Furthermore, there is an accumulating series of reports that link altered GIT microbial profiles to behavior and adverse mood states [86]. The continuous intake of fermented milk containing probiotics has been reported to positively modulate behavior in healthy adults [87]. This study suggests that metabolites produced by bacterial fermentation may be important in behavior.

Interestingly, a recent report has demonstrated that some genera of human GIT bacteria can induce a rapid increase of reactive oxygen species that then elicit a physiological response through the activation of epithelial NAPDH oxidase-1 (Nox1) [76,88]; we have reported the detailed mechanism of ROS activity in the gut elsewhere [89].

For an in-depth appraisal of this section see [23,90,91,92,93].

### 3.4. Metabolites that Risk Disrupting GIT Host-Microbial Homeostasis 

The microbiota of the human gut assists the human host with beneficial activities such as reducing the risk of infections and resisting allergies [23]. However, foods that disrupt the homeostasis of the GIT microbiota can increase the risk of diseases including obesity, inflammatory bowel diseases, and cardiovascular disease [94]. For example, a recent murine study has reported that the emulsifier ingredients that contribute to the smooth and stable consistency of foods such as ice cream and chocolates may promote certain chronic inflammatory diseases [95]. The triggered adverse inflammatory response was posited to result from a negative influence on the GIT microbiota. 

Furthermore, gut microbial metabolic activity in mice and humans of dietary food-derived compounds such as phosphatidylcholine and L-carnitine has been reported to generate metabolites such as trimethylamine, which is further metabolized to trimethylamine-*N*-oxide, a proatherogenic agent that increases the risk of disease [96,97]. A high fat diet has been postulated to increase the risk of hepatocellular carcinoma due to an altered gut microbial composition [98]. The mechanism implicated an uncontrolled increase in deoxycholic acid-producing bacteria from the cluster XI of the genus *Clostridium.* Deoxycholate is a secondary bile salt that can induce phenotypic changes in hepatic stellate cells leading to oversecretion of pro-inflammatory cytokines that can facilitate the development of a hepatocellular carcinoma. Other experimental observations propose that metabolites elaborated by the GIT microbiome such as p-cresol, indolyl-3-acryloylglycine, and indole pyruvate can also alter behavior [99]. Recently it has been posited that the gut may be the primary site of uremic toxin production, which is linked to chronic kidney disease [100]. Therapies with probiotics and prebiotics may have a therapeutic role in re-establishing a balanced GIT that reduces the progression of chronic kidney disease and its associated uremia. A recent review has postulated that uremic toxins such as indoxyl sulphate can be generated by the exaggerated activity and overgrowth of pathobionts, which leads to gut dysbiosis and increased circulating uremic toxins that eventually leads to adverse effects on the kidneys [101].

For an in-depth appraisal of this section see [102,103,104].

### 3.5. GIT Bacteriophages Chemical Modulators of the GIT Microbiome

Bacteriophages (designated as bacterial viruses or phages) have been reported to comprise the most abundant replicating entities on planet earth with an estimated population size of ≥10^30^ phage particles [105,106,107]. This impression has been supported by findings that indicate that total viral abundance exceeds that of total prokaryotic abundance in aquatic systems by a factor of 10 [108]. Recently it has been hypothesized [109] that bacteriophages can shape the mammalian GIT microbiome. Therefore, given that phages are relatively specific, have the capacity of rapid adaptation, and can often be an obligate terminator of a host cell’s life cycle, they can impose strong selection on bacterial populations and hence shape microbial communities [110].

Prophages can significantly alter the GIT microbial community composition, and that in turn leads to the induction of intestinal dysbiosis by altering the ratio of symbionts to pathobionts. The mechanism proposed is the enabling of pathobiont niche re-establishment in the GIT.

The human GIT [111] has been reported to harbor approximately 10^15^ bacteriophages. Due to their phylogeny diversity and variation, bacteriophages have been postulated to participate both in health and disease, in the maintenance of gut homeostasis or in the development of inflammatory bowel conditions and diseases. The major phages that have been most studied and hence reported are those from the *Siphoviridae, Myoviridae, Podoviridae*, and *Microviridae* groups [112,113]. In a recent review [111] it was noted that the phages from these groups overlapped between healthy adults and those with a diagnosis of inflammatory bowel disease, indicating that in healthy adults there was high phage diversity and stability as compared to adults with Crohn’s disease, who had low diversity.

The exact origin of the phages in the human gut remains largely unidentified. It has been postulated that the most likely sources of bacteriophages are nutritional practices and the diet, as well as the environment through the induction of prophages from the microbiota themselves, or from the mother [111]. It is of notable significance that even the most abundant viral sequences in a healthy infant were not detected in either breast milk or formula [113]. Environmental factors have been reported as triggers for virion variations detected in the saliva samples of unrelated individuals living in the same household [114]. 

For an in-depth appraisal of this section see [110,111].

## 4. Discussion

All coelomate vertebrates and invertebrates have co-evolved with symbiotic gut microbial species that perform multiple digestive and metabolic functions for the host.

The resultant capacity of microbial organisms to participate in enhanced adaptive mechanisms for energy generation is an example of how these micro-organisms exploit their immediate environment for new dietary resources. This activity offers a real-time opportunity to elucidate novel compounds that comprise the language of bacteria. For example, numerous small molecules that have been termed ribosomally synthesized, post-translationally modified peptides have been reported to have a narrow spectrum of activity (e.g., lantibiotics, microcins) [10]. The biological relevance of this restricted activity may indicate that these molecules may have inherent signaling abilities to control the local environment that the bacteria occupy. 

The administration of a multi-species probiotic is, in principle, a biologically plausible challenge to exploit the beneficial effects of the commensal microbiota in favor of the host. In most instances, the molecular underpinning of these exchanges remains to be defined but represents an intriguing strategy for identifying therapeutic targets and pharmacotherapeutic discovery.

While the therapeutic potential of immunomodulatory molecules from pathogens is recognized, the pursuit of elucidating bioactive molecules from the commensal gut microbiota is a more recent development. Classical examples include the discovery of bacteriocins that demonstrate a strong anti-bacterial action against *Clostridium difficile* as well as bacterial-derived immunomodulatory molecules. Other compounds such as SCFAs and microbe-associated molecular patterns are important microbial signals that the host is able to detect and respond to. These entities can have an overlapping influence on GIT active tissues such as the gut epithelium, neuroendocrine, and lymphoid cells that orchestrate and fine-tune homeostasis, as evidenced by maintaining balanced local pro- and anti-inflammatory tone, satiety/energy balance, and GIT motility functions.

## 5. Conclusions

Mechanistic insight into the activities of the microbiota while deciphering the specific molecular language used will advance the molecular understanding of mechanisms of crosstalk between the host and microbe and between microbes. This will assist in elucidating their role in health and disease. Furthermore extending this critical thinking should include the role of bacteriophages and their origins, as phage interactions may demonstrate an acquired individuality profile that is based on diet and environmental/geographical conditions encountered. As such, they could also feasibly contribute a marker for health and disease.

In humans, early life prudent interactions with dietary preferences, lifestyle, and genetic make-up influence microbial populations (and phage populations) that significantly contribute to ecological development (the milieu). These interactions may well determine the health of the host, which is then translated to individuals and populations as well as the diversity of the microbiomes and phage cohorts that inhabit them.
